# Domain-Generality of Timing-Based Serial Order Processes in Short-Term Memory: New Insights from Musical and Verbal Domains

**DOI:** 10.1371/journal.pone.0168699

**Published:** 2016-12-19

**Authors:** Simon Gorin, Benjamin Kowialiewski, Steve Majerus

**Affiliations:** 1 Psychology and Neuroscience of Cognition Research Unit (PsyNCog), Faculty of Psychology, Speech Therapy and Educational Sciences, University of Liège, Liège, Belgium; 2 Fund for Scientific Research–FNRS, Brussels, Belgium; Katholieke Universiteit Leuven, BELGIUM

## Abstract

Several models in the verbal domain of short-term memory (STM) consider a dissociation between item and order processing. This view is supported by data demonstrating that different types of time-based interference have a greater effect on memory for the order of to-be-remembered items than on memory for the items themselves. The present study investigated the domain-generality of the item *versus* serial order dissociation by comparing the differential effects of time-based interfering tasks, such as rhythmic interference and articulatory suppression, on item and order processing in verbal and musical STM domains. In Experiment 1, participants had to maintain sequences of verbal or musical information in STM, followed by a probe sequence, this under different conditions of interference (no-interference, rhythmic interference, articulatory suppression). They were required to decide whether all items of the probe list matched those of the memory list (item condition) or whether the order of the items in the probe sequence matched the order in the memory list (order condition). In Experiment 2, participants performed a serial order probe recognition task for verbal and musical sequences ensuring sequential maintenance processes, under no-interference or rhythmic interference conditions. For Experiment 1, serial order recognition was not significantly more impacted by interfering tasks than was item recognition, this for both verbal and musical domains. For Experiment 2, we observed selective interference of the rhythmic interference condition on both musical and verbal order STM tasks. Overall, the results suggest a similar and selective sensitivity to time-based interference for serial order STM in verbal and musical domains, but only when the STM tasks ensure sequential maintenance processes.

## Introduction

Language and music share the characteristic of being both composed of complex auditory structures unfolding over time. Although the extent to which verbal and musical information processing rely on similar neurocognitive resources and mechanisms is still a matter of debate [1–4], recent studies indicate the existence of a close link between linguistic and rhythmic abilities [5–11], and suggest the existence of common timing-based processes involved in language and music. This is supported by the fact that children with better perceptual rhythmic skills have better morpho-syntactic production [8] as well as by the observation of a link between reading abilities and meter perception and rhythm processing [7]. These studies are in line with the *resource-sharing framework* proposed by Patel [12], positing that the processing of verbal and musical information, while relying on different domain-specific sensory representations, share common neural resources when involving similar cognitive tasks. One of the domain-general mechanisms tapped by these similar cognitive tasks is considered to be auditory short-term memory (STM), which serves to maintain sequential and temporally organized auditory information [13–15]. At the same time, there is evidence showing that amusic participants have impaired STM capacities for pitch information while keeping STM capacities for verbal material preserved [16], indicating that STM for verbal and musical stimuli do not always overlap [17,18]. The aim of this study is to shed further light on the similarities and dissimilarities of STM in verbal and musical domains, by making a critical distinction between item and serial order STM processes.

In the verbal STM (vSTM) domain, an important feature of many theoretical models is the distinction between item-based and serial order-based retention processes (e.g., [19–25]. Some of these models consider that a list of memoranda is represented in memory by the associations between item representations, activated in long-term memory, and a dynamic context-signal or episodic token representation, for the representation of serial order information (e.g., [20,21,23]. This distinction stems from the observation that psycholinguistic factors reflecting linguistic knowledge stored in long-term memory influence recall of item identity (item information) to a stronger degree than recall of the serial position of the items within in a list (order information) [26–30]. There is also evidence that other factors can disrupt selectively STM performance for order relative to item information. When STM tasks are performed concurrently with interfering tasks (e.g., irrelevant speech, irrelevant tones, articulatory suppression, finger tapping), STM performance for serial order information is impacted to a greater extent than is maintenance of item information [31–34]. The selective effect of irrelevant auditory distractors on order STM has been interpreted as a conflict between concurrent seriation processes involved in the maintenance of order information in STM as well as in the processing of distracting auditory information [35,36]. Other studies proposed that this effect of interference is due to the involvement of similar motor planning programs in the interfering and serial order recall tasks [37,38], which is also consistent with the serial conflict hypothesis considering that similar serial order mechanisms are involved in memory for order and control of speech production [39]. Conversely, while these effects of interference have been mainly shown in the field of vSTM, there is also evidence that reproduction of non-verbal, rhythmic patterns from STM is disrupted by concurrent verbal articulatory suppression tasks [40], supporting the involvement of similar, and possibly temporal processes in the two tasks. This is also in line with a study showing an association between the size of the memory span (verbal and visual) and STM capacity for rhythmic patterns [41].

One of the few studies directly studying the hypothesis of time-based signals for coding order information in STM, by further distinguishing between item and serial order STM processes, is the study by Henson et al. [31]. The authors showed that recognition performance in item probe tasks is impacted to a lesser extent by interfering tasks thought to involve a timing-signal component (e.g., articulatory suppression or rhythmic tapping) than is performance in list probe tasks, assessing STM for serial order information. In the light of these results, the authors proposed that STM for serial order is supported by a timing-signal process (see [20,23], and that irrelevant speech, articulatory suppression and finger tapping share a temporal component, recruiting the same timing-signal processes as STM for serial order. A number of theoretical models of vSTM consider that serial order information is represented via temporal signals, in the form of a changing episodic context signal or clock-like internal oscillators [20,23]. This is also supported by recent studies showing that temporal grouping effects in vSTM, which occur when items are presented in rhythmically organized sequences, can be explained by multi-oscillator models of vSTM [42]. Hartley et al. [42] assume that these oscillators encode the timing and the rhythmic organization of the to-be-remembered items in a sequence, where timing-related information is extracted based on phase and amplitude signal-changes of the speech envelope.

This assumption of vSTM for serial order information relying on temporally organized representational processes attributes the representation of serial order information to domain-general event-driven timing processes [42] which could also serve to store serial order information in other modalities characterized by temporally organized information, such as musical sequences. Williamson et al. [43] proposed that, while relying on distinct representational stores for item information processing [44], serial order processing in vSTM and musical STM (mSTM) could rely on similar rehearsal processes. There are indeed a number of phenomena in the musical domain which resemble those observed for serial order processing in vSTM [45–47]. First, a strong preponderance of serial ordering errors is observed in both vSTM recall tasks and musical sequence production tasks (about 80%) [45–48]. Secondly, in these two types of tasks, serial ordering errors tend to conform to a *locality constraint*, where the probability of serial position exchange errors decreases when serial position displacements increase in size [45–47,49–51]. Thirdly, in the musical domain the probability of serial position exchanges between items from different musical segments increases for items having the same metrical signature within each segment [45]. This is similar to the temporal grouping effect observed in vSTM, where between-group item migrations tend to keep the same within-group serial position [52–54].

The aim of the present study is to further investigate the existence of domain-general principles in vSTM and mSTM, by determining to what extent the dissociation between item-based and serial order-based STM processes observed in the verbal domain also applies to the musical domain, and by determining to what extent serial order STM is sensitive to time-based interference effects in both verbal and musical domains. This is based on the hypothesis developed here that processing of item information in the vSTM and mSTM domains is supported by domain-specific representational structures while processing of serial order information relies on domain-general event-driven timing processes. Specifically, we will present different STM tasks manipulating the abilities to retain either item information or serial order information, this for each domain (verbal and musical), and under conditions of no-interference or conditions of interference containing a temporal component. We expected that time-based interfering tasks will lead to a stronger interfering effect on maintenance of serial order information than on maintenance of item information in both domains, in line with Henson et al. [31].

## Experiment 1

The present experiment pursues two principal aims: 1) exploring the dissociation between item and order information processing in mSTM, and 2) exploring the domain-generality of event-driven timing processing of serial order information for vSTM and mSTM. We conducted different musical and verbal short-term recognition tasks manipulating item or order STM retention requirements; each task was administered under different conditions of interference, the secondary interfering tasks being presented during the maintenance phase of each task (no-interference: silent maintenance phase; rhythmic interference: maintenance phase filled by an unpredictable rhythm to which participants had to react by finger-tapping response; articulatory suppression: participants have to continuously produce the syllable “bla” during the maintenance phase). The STM tasks required listeners to make same/different judgments between pairs of four-item sequences. In the item STM task, the participants had to maintain and recognize the identity of items presented, independently of their serial order of occurrence; in the order STM task, the participants had to maintain and recognize the serial order of occurrence of the items within a sequence. We hypothesized that both interfering tasks will impact more vSTM for order information than vSTM for item information, in line with Henson et al. [31]. If serial order processing in mSTM is supported by the same mechanisms as in vSTM, the interfering tasks should produce the same pattern of interference in both modalities. The continuous and regular repetition of syllables during the articulatory suppression interfering task and the reproduction of a rhythmic sequence during the rhythmic interfering tasks require access to temporal encoding and control processes, and hence are thought to compete for the same timing-signal as the serial order STM task [20,55]. Henson et al. [56] showed that processing of information during serial order STM tasks recruited the same premotor brain regions as those involved in the reproduction of temporal information, such as during reproduction of rhythmic finger movements.

### Materials and Method

The study has been approved by the local Ethics Committee of the Faculty of Psychology, Speech and Language Therapy, and Education from the University of Liège, and all participants gave their informed written consent before starting the experiment.

#### Participants

Thirty-six participants, all being French Belgian native speakers, (M_age_ = 22.4 years, SD = 3.6) participated in the present experiment, with on average 1.4 years of musical practice (SD = 1.8), 0.3 years of formal music theory lesson (SD = 1.1); all had a high educational level (M = 14.1 years, SD = 1.5).

#### Stimuli

The stimuli for the vSTM tasks were based on a set of nonwords used by Majerus et al. [57] in a previous experiment. The set was composed of 60 easily pronounceable disyllabic nonwords subdivided in 30 minimal pairs differing only by a consonant (mean nonword duration = 573 milliseconds, SD = 92). Note also that nonword pairs were matched for digram and diphone segments occurrences in French (for more details, see [57]) and had been recorded by a French-speaking Belgian native male speaker. For the mSTM tasks, the stimuli consisted of a set of 20 tones (from C_2_ to A_5_) covering four octaves and arranged according to a major pentatonic scale. In each octave we selected five tones corresponding to a C major scale omitting the fourth and the seventh scale degrees, thus forming a major pentatonic scale. By doing this with four consecutive octaves we obtained a set of 20 tones organized around a C major pentatonic scale. This allowed us to construct tonally structured sequences which are not too familiar; note that the verbal stimuli used in this experiment, nonwords, were also unfamiliar. The tones were generated with Anvil Studio 2011 (version 2011.09.06) as MIDI files using a piano timbre, then converted in.wav format and normalized to a duration of 800 milliseconds with a rise and fall period of 10 milliseconds. The rhythmic interference sequences were created based on a unique cross-stick drum sound timbre lasting for 25 milliseconds that was generated with Guitar Pro (version 6) as.wav file; half of the sequences were composed of six drum sounds and the other half of seven drum sounds in order to avoid that participants could predict the number of beat pulse occurrences in the rhythmic sequences.

#### Design

**Fig 1:**
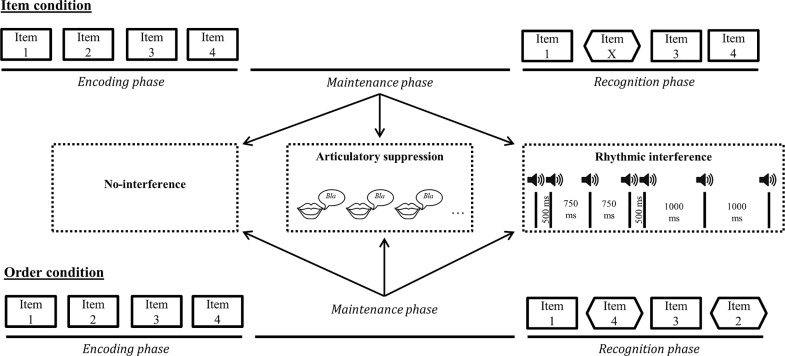
Graphical representation of task design for Experiment 1. Examples represent non-matching sequence probes for each STM task condition (i.e. item and order). Note that the same task setup characterized matching probe trials. All the task conditions (verbal *versus* musical and item *versus* order) were performed under no interference, articulatory suppression, or rhythmic tapping interference conditions.

The experiment consisted of 192 trials. The trials were spread into 12 blocks each composed of 16 trials, following a 2 × 2 × 3 experimental design with a stimulus domain factor (verbal and musical), a STM task condition factor (item and order), and an interference type factor (no-interference, articulatory suppression, and rhythmic interference). See Fig 1 for an overview of the experimental design.

For the vSTM trials, the memory lists were created by pseudo-randomly selecting four different items in the nonword set. We ensured that no minimal pairs of items (e.g., /pe**m**al/ and /pe**g**al/) were in the same memory list and that all items inside a sequence began with a different consonant. We also ensured that all sequences were unique over all blocks.

For the mSTM trials, the memory lists were created by randomly selecting four different tones from the two lower octaves and the other half from the two higher octaves. We also ensured that within each musical block, all pitch classes (i.e., C, D, E, G, A) occurred equally often among the four serial position, and that all sequences were unique over all blocks.

For the rhythmic interference task, the stimulus onset asynchronies (SOAs) were pseudo-randomly selected among three different SOAs (500, 750 and 1000 milliseconds), with the last SOA defining the interval between the last beat pulse and the first item of the comparison sequence. These SOA values were specifically selected to create rhythmically irregular and unpredictable sequences, while ensuring that the inter-onset interval between the first beat pulse of the rhythmic sequence and the first item of the probe sequence was always of 4500 milliseconds.

#### Procedure

The stimuli were presented through headphones connected to a mobile workstation. The participants heard the memory list (SOA: 1 second), followed by a five-second maintenance phase after which the probe sequence was presented. The participants made a same/different judgment by pressing one of two response buttons. Task instructions were given before each block, and the two first trials were practice trials. For both vSTM and mSTM tasks, half of the trials were non-matching trials. In the vSTM task, the probe sequence mismatched the memory list by exchanging a single phoneme (central consonant) of one of the four nonwords (item condition), or by a exchanging the serial positions of two non-adjacent items (half of the changes were near exchanges and were separated by two positions, e.g. positions 1–3 or positions 2–4, and the other half of changes were distant exchanges separated by three positions, e.g., positions 1–4). Note that while order vSTM tasks usually involve adjacent changes [31,57–59], we used non-adjacent serial position exchanges in order to enable us to preserve the overall contour pattern of target and probe sequences for the musical stimuli. For the musical stimuli, the probe sequence mismatched the memory list by changing, in the item condition, the pitch of a single tone to the pitch of the next highest or the next lowest tone in the pentatonic scale (e.g., D in a sequence could be replaced by C or E if these tones were not already present in the target sequence), or by exchanging, in the serial order condition, the serial positions of two non-adjacent tones.

The 12 blocks were presented in a counterbalanced order and participants were informed about the block condition before the beginning of each block to ensure that task requirements were known in advance. Trials inside each block were presented in a random order. For the item conditions, participants were instructed to decide whether all the items in the memory list matched those of the probe list. For the order conditions, participants were instructed to decide whether the serial position of the items in the probe sequence matched those of the memory list. For blocks with the articulatory suppression interference condition, participants were asked to continuously repeat the syllable “bla” during the five-second maintenance phase. For the rhythm interference blocks, participants had to react by finger tapping response to each beat occurrence of an auditory rhythmic sequence starting 500 milliseconds after the memory list and stopping 500, 750 or 1000 milliseconds before the presentation of the probe list, this as a function of the SOA value of the last beat pulse of the rhythmic sequence.

Task presentation was controlled by the software Opensesame (version 2.8.3, [60]). At the end of the experiment, participants were required to fill out a questionnaire composed of anamnestic questions and questions related to the strategies used during the different tasks.

#### Statistical Analysis

In accordance with recent recommendations [61–66], we conducted Bayesian analyses, in addition to frequentist univariate analysis, using the *anovaBF* function of the *BayesFactor* [67] package run in *R* [68] with default settings. Frequentist inferential statistics are problematic due to the use of *p*-values and associated estimation bias in favor of H_1_ (e.g., [66]. One solution is to use a model selection method, as proposed by Bayesian techniques, allowing to compare different models and to quantify the strength of evidence that the data provide for each specific model. Bayesian methods have the advantage that they allow the evidence for *and* against the null hypothesis to be quantified, whereas classical inference methods can only provide evidence against, but not for, the null hypothesis [61].

For the Bayesian analysis we followed the decision criterions proposed by Lee and Wagenmakers [69] considering a Bayes Factor (BF) of < 3 as anecdotal evidence, between three and 10 as moderate evidence, between 10 and 30 as strong evidence, between 30 and 100 as very strong evidence, and higher than 100 as decisive evidence for the model tested relative to the null model or relative to another model.

### Results

Analysis of raw data showed that one participant had extreme values in the item verbal condition, with task accuracy lower than .50 already in the no-interference condition and representing performance lower than two standard deviations below the group mean. To avoid biasing data by these values, we removed the participant from all the following analyses.

#### Recognition Performance

**Fig 2:**
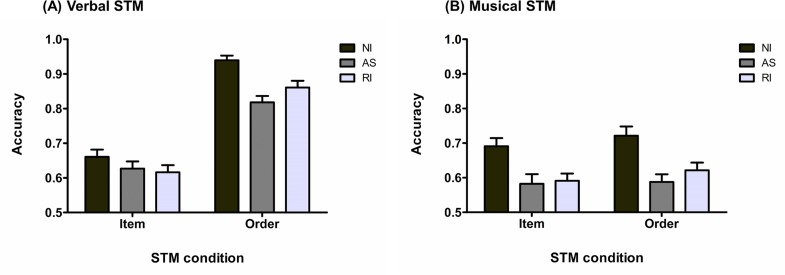
Response accuracy for Experiment 1. (A) Response accuracy (mean and standard error) for the vSTM task as a function of STM task and interference conditions (NI: no-interference; AS: articulatory suppression; RI: rhythmic interference). (B) Response accuracy (mean and standard error) for the mSTM tasks as a function of STM task and interference conditions (NI: no-interference; AS: articulatory suppression; RI: rhythmic interference).

A 2 × 2 × 3 repeated measures analysis of variance (rmANOVA) on the mean proportion of correct response revealed, as shown in Fig 2, that the three main effects were significant, with an effect of stimulus domain, *F*_(1, 34)_ = 66.27, *p* < .001, MSE = .02, η_p_^2^ = .66, of STM task condition, *F*_(1, 34)_ = 205.04, *p* < .001, MSE = .01, η_p_^2^ = .86, and of type of interference, *F*_(2, 68)_ = 37.50, *p* < .001, MSE = .01, η_p_^2^ = .52. These results indicate an effect of domain expertise with significantly better performance for the verbal condition relative to the musical condition, as well as an effect of STM task condition with higher performance for the order condition. Regarding the effect of interference, both interfering tasks led to lower performance relative to the no-interference condition (Tukey’s post hoc for the effect of interference, with alpha = .05: no-interference *versus* articulatory suppression, *p* < .001; no-interference *versus* rhythmic interference, *p* < .001; rhythmic interference *versus* articulatory suppression, *p* = .28). The only significant interaction was the stimulus domain-by-STM task condition interaction, *F*_(1, 34)_ = 85.10, *p* < .001, MSE = .01, η_p_^2^ = .71. Tukey’s post hoc analysis (alpha = .05) revealed that the interaction was characterized by a significant difference between the item and order task conditions in the verbal domain (*p* < .001) but not in the musical domain (*p* = .55). All others interactions were non-significant: auditory domain-by-type of interference, *F*_(2, 68)_ = 1.51, *p* = .23, MSE = .01, η_p_^2^ = .04, STM task condition-by-type of interference, *F*_(1.71, 58.04)_ = 2.44, *p* = .10, MSE = .01, η_p_^2^ = .07, and three-way interaction, *F*_(2, 68)_ = 0.73, *p* = .48, MSE = .01, η_p_^2^ = .02.

Bayesian rmANOVA showed that the model with the highest BF was the model including the main effects of stimulus domain, STM task condition, interference type and the stimulus domain-by-STM task condition interaction (BF = 3.71E+55). This model was preferred over the same model including the STM task condition-by-type of interference interaction by a factor of 2.83, which was the model with the next highest BF.

#### Response Latencies

**Fig 3:**
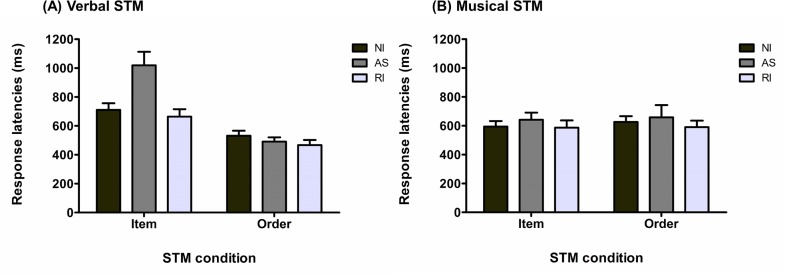
Response latencies for Experiment 1. (A) Response latencies (mean and standard error) for the vSTM task as a function of STM task and interference conditions (NI: no-interference; AS: articulatory suppression; RI: rhythmic interference). (B) Response latencies (mean and standard error) for the mSTM task as a function of STM task and interference conditions (NI: no-interference; AS: articulatory suppression; RI: rhythmic interference).

Next we analyzed response latencies for correct responses (see Fig 3). Note that two participants were discarded from this analysis due to response latencies (RL) higher than three standard deviations above the group mean for the musical condition for the first participant and higher than three standard deviations above the group mean for the verbal condition for the second participant. We performed a 2 × 2 × 3 rmANOVA on the mean of the median correct RL across participants. We obtained a significant main effect of STM task condition characterized by slower RL in the item STM task condition, *F*_(1, 32)_ = 25.56, *p* < .001, MSE = 7.79E+4, η_p_^2^ = .44, and of type of interference, *F*_(1.58, 50.69)_ = 6.42, *p* = .006, MSE = 8.46E+4, η_p_^2^ = .17, but there was no significant main effect of stimulus domain, *F*_(1, 32)_ = 0.91, *p* = .35, MSE = 1.06E+5, η_p_^2^ = .03. Tukey’s post hoc analysis (alpha = .05) revealed that for the effect of interference only the articulatory suppression led to significantly slower RL, and this relative to the two other conditions (articulatory suppression *versus* no-interference: *p* = .05; articulatory suppression *versus* rhythmic interference: *p* = .002; no-interference *versus* rhythmic interference: *p* = .53). Finally, all the two-way interactions as well as the three-way interactions were significant (STM task condition-by-stimulus domain: *F*_(1, 32)_ = 21.90, *p* < .001, MSE = 1.15E+5, η_p_^2^ = .41; STM task condition-by-type of interference: *F*_(2, 64)_ = 6.84, *p* = .002, MSE = 4.72E+4, η_p_^2^ = .18; stimulus domain-by-type of interference: *F*_(2, 64)_ = 3.44, *p* = .04, MSE = 4.27E+4, η_p_^2^ = .10; three-way interaction: *F*_(1.33, 42.50)_ = 4.26, *p* = .03, MSE = 7.39E+4, η_p_^2^ = .12) The three-way interaction was decomposed by running separate rmANOVAs as a function of the stimulus domain condition.

For the musical stimulus domains (see Fig 3A), a 2 × 3 rmANOVA showed no significant main effects or interaction (STM task condition: *F*_(1, 32)_ = 0.16, *p* = .69, MSE = 9.58E+4, η_p_^2^ < .001; type of interference: *F*_(1.69, 54.13)_ = 1.14, *p* = .32, MSE = 5.52E+4, η_p_^2^ = .03; two-way interaction: *F*_(1.68, 53.89)_ = 0.05, *p* = .93, MSE = 6.34E+4, η_p_^2^ < .01). This result was confirmed by a Bayesian rmANOVA showing that the null model containing only the random effect of participant was preferred to the model with the highest BF, which included only a main effect of STM task condition, by a factor of 5.89; this indicates that the null model is moderately more probable for the musical domain.

To the opposite (see Fig 3B), all the effects were significant in the verbal domain. A 2 × 3 rmANOVA showed a main effect of STM task condition with faster RL for the order condition, *F*_(1, 32)_ = 46.33, *p* < .001, MSE = 9.70E+4, η_p_^2^ = .59, and effect of type of interference, *F*_(2, 64)_ = 8.69, *p* < .001, MSE = 7.21E+4, η_p_^2^ = .21; Tukey’s post hoc analysis (alpha = .05) indicated that only the articulatory suppression condition significantly slowed RL relative to the two other conditions (articulatory suppression *versus* no-interference: *p* = .02; articulatory suppression *versus* rhythmic interference: *p* < .001; no-interference *versus* rhythmic interference: *p* = .50). Tukey’s post hoc analysis (alpha = .05) conducted on the significant two-way interaction, *F*_(2, 66)_ = 11.00, *p* < .001, MSE = 5.77E+4, η_p_^2^ = .26, indicated no significant difference between the three types of interference in the order condition (no-interference *versus* articulatory suppression: *p* = .98; no-interference *versus* rhythmic interference: *p* = .88; articulatory suppression *versus* rhythmic interference: *p* = .99), while articulatory suppression significantly slowed RL in the item STM condition relative to the two other interference conditions (articulatory suppression *versus* no-interference: *p* < .001; articulatory suppression *versus* rhythmic interference: *p* < .001; no-interference *versus* rhythmic interference: *p* = .97). Bayesian rmANOVA decisively supported these results. Analyses revealed that the model explaining the data best was the full model (BF = 8.12E+12), which was preferred over the second model with the highest BF containing only the main effects by a factor of 137.84.

#### Analysis of Strategies

A final set of analyses focused on the use of strategies used by the participants. As shown in Table 1, 97% of participants reported having used verbal auditory rehearsal strategies during vSTM tasks while only 51% of participants reported having used some kind of musical auditory rehearsal strategy during the mSTM tasks. At the same time, 91% of participants reported to rely also on visual strategies–visualization of graphical contour shape–for the maintenance of musical sequences while this was the case for only 15% of participants in the verbal auditory domain. Note also that this analysis was conducted on only 33 participants since no information about maintenance strategies could be obtained for the two first participants.

**Table 1:** Distribution of maintenance strategies used in Experiment 1 as a function of the STM domains.

Verbal domain		Musical domain	
Rehearsal strategies	Distribution	Rehearsal strategies	Distribution
Auditory	81.82%	Auditory	9.09%
Visual imagery	3.03%	Graphical imagery	27.27%
Auditory + visual imagery	15.15%	Up-down contour imagery	21.21%
		Auditory + graphical imagery	21.21%
		Auditory + up-down contour imagery	21.21%

### Discussion

The present experiment aimed at testing the hypothesis that vSTM and mSTM are supported by domain-general event-driven timing processes, and this specifically for the representation of serial order information as compared to item information. Overall, the results obtained are not strongly in favor of a selective sensitivity of serial order STM abilities to tasks involving temporal interference, in neither verbal nor musical STM domains, and contrast with the results obtained by Henson et al. [31] in the verbal domain.

First of all, performance was more accurate and faster for the serial order STM than the item STM condition, and this particularly in the verbal domain, while serial order STM tasks generally lead to lower accuracy and longer reaction times due to the serial scanning processes involved in this task [31]. This unexpected result may have been due to the use of specific strategies in a number of our participants. Indeed, 15 and two out of 33 participants reported remembering only the first letter or the last consonant of the nonwords, respectively, in the verbal serial order STM conditions, leading to an overall lower amount of stimulus information to be processed in the serial order STM task. This strategy could not be used in the verbal item condition, given that mismatching items involved a change of consonant in middle positions of one of the nonwords; hence, participants needed to process and remember the whole items in order to make correct recognition judgments. This may also explain the significantly increased RL for the verbal item STM task under the articulatory suppression, where the verbal content of the articulatory suppression is likely to have interfered with the maintenance of verbal item information.

Furthermore, contrary to our predictions, the interfering tasks had the same deleterious effect on recognition performance for both item and order information in verbal and musical domains, and this particularly for response accuracy. For RL, except for the increased RL for the verbal item STM condition under articulatory suppression already discussed in the previous paragraph, no significant effect of interference was observed. These results are in striking contrast to those observed by Henson et al. [31]. Again, one of the reasons explaining these discordant findings could be related to the first-letter encoding strategy used by participants in the verbal order STM conditions, overall diminishing STM load for the serial order STM conditions, and hence also leading to a smaller interference effect as could have been obtained if participants had processes all stimuli to the same extent as in the item STM conditions. This argument cannot account for the absence of STM condition specific interference effects in the mSTM modality. However, in the mSTM modality, participants reported using mainly visual, contour-based maintenance strategies (see Table 1) and this for both item and serial order STM conditions, which could have diminished the sequential encoding and maintenance processes especially in the musical serial order STM conditions. If participants make recognition judgment of the melodies based on a visuo-spatial representation of the contour shape of the melodies, this will result to a large amount of non-detections of non-matching trials for both item and order mSTM tasks, given that both item and order mSTM tasks involved the presentation of non-matching sequences that did not violate the contour of the target sequence. This was indeed supported by the observation of a high rate of false alarms in both item and order mSTM conditions (item no-interference: .44; item articulatory suppression: .51; item rhythmic interference: .60; order no-interference: .39; order articulatory suppression: .52; order rhythmic interference: .56). More generally, Macken et al. [70] stated that recognition tasks may rely to a greater extent on the use of perceptual global matching strategies than do recall tasks, and hence may not the best suited to assess dissociations between different types of information stored in STM.

Given the use of clearly different strategies in the verbal and musical STM tasks as well as in the verbal item and order STM conditions, the results observed in Experiment 1 and their implications for a dissociation of item and serial order STM processes in vSTM and mSTM are difficult to interpret in an unambiguous manner. Experiment 2 controlled for these differences in strategy use by using a more constrained STM task. Furthermore, in order to make our experimental design more directly comparable to the one used in the study by Henson et al. [31], we only used a single interference condition in Experiment 2 since Henson et al. [31] also only used one interference condition in each of their experiments. The cognitive load involved in switching between different secondary tasks in a single experiment may have further led participants to circumvent STM task difficulty by using non-sequential, global maintenance strategies in mSTM modality and first-letter encoding strategies in the vSTM modality. In Experiment 2, we retained the rhythmic interference condition only, this for several reasons. First, the rhythmic interference task requires more fine-grained and unpredictable temporal order analyses and reproduction than the articulatory suppression condition, and hence is the theoretically most informative interference condition, relative to articulatory suppression (see also Henson et al. [31]). Furthermore, the rhythmic interference task uses more neutral information regarding the two auditory domains of interest, given that the rhythmic beats are distinct at a representational level from both the nonwords and tones, while the syllables to be repeated in the articulatory suppression task had a strong verbal component potentially overlapping with the nonwords of the verbal condition, as suggested by the strongly increased RL for the verbal item STM condition carried out under articulatory suppression.

## Experiment 2

In Experiment 2, in order to avoid the use of global-matching procedures and first-letter encoding strategies, participants were required to process information in a full sequential manner during encoding, maintenance and recall via a sequential recall and recognition STM procedure. The task design of Experiment 2 involved the presentation of sequences of CV-structure syllables or tones presented in time with a regular beat sequence. After a maintenance phase, participants were required to covertly reproduce the sequence synchronously with the beat sequence. One of the beats was accompanied by the presentation of an item (syllable or tone), and the participants had to determine whether the item matched the nature of the item of the corresponding beat in the target sequence (item task), or whether the item has been presented in the corresponding serial position in the target sequence (serial order task).

Furthermore, in Experiment 2, serial position exchanges for non-matching trials were limited to adjacent serial positions in order to test serial order coding in the most sensitive manner given that serial order exchange errors in recall follow a *locality constraint* [49,50] with the strongest error rate for adjacent serial positions. In Experiment 1, only non-adjacent serial position exchanges had been used.

### Materials and Method

The study has been approved by the local Ethics Committee of the Faculty of Psychology, Speech and Language Therapy, and Education from the University of Liège, and all participants gave their informed written consent before starting the experiment.

#### Participants

Thirty participants (M_age_ = 22.8 years, SD = 3.5) took part in this second experiment based on a voluntary basis, all participants were native French Belgian speakers except for one participant who was a German-French bilingual speaker. They had on average 0.3 years of formal musical theory instruction (SD = 1.1), and 3.0 years of musical practice (SD = 4.5), and all had a high level of education (M = 15.6 years, SD = 3.0). Participants were asked about their hearing status and no participants claimed having absolute pitch or reported specific hearing impairment.

#### Stimuli

For the verbal STM tasks, the stimuli were CV mono-syllabic stimuli instead of the bi-syllabic stimuli used in Experiment 1. This was done in order to equate STM load and task difficulty for the item and order conditions and further reduce the likelihood that participants only encoded the initial letter of the stimuli. We selected 25 CV-syllables constructed from a pool of five consonants (B, D, P, T, V) and a pool of five vowels (A, E, I, O, U); the syllables had been recorded by a French-speaking Belgian native female speaker (mean syllable duration = 259 milliseconds; SD = 57). The stimuli used for the mSTM tasks were seven tones of a C major scale (from C_2_ to B_2_, included) created with Anvil Studio 2011 (version 2011.09.06) as 500 milliseconds MIDI files using a piano timbre; they were converted in.wav format with a rise and fall period of 10 milliseconds. Finally, the rhythm interference sequences were created based on a unique cross-stick drum sound lasting for 25 milliseconds generated with Guitar Pro (version 6) and stored as.wav file, as in Experiment 1.

#### Design

The experiment consisted of 128 trials separated in eight blocks of 16 trials according to a 2 × 2 × 2 experimental design, with a stimulus domain factor (verbal and musical), a STM task condition factor (item and order), and a type of interference factor (no-interference and rhythmic interference).

For the vSTM tasks, each memory list consisted of four-syllable memory sequences created by pseudo-randomly selecting four pairs of consonants and vowels in the set (e.g., [B, T, P, D] + [O, I, A, U] = [BO, TI, PA, DU]), while the remaining pair (in that case VE) was used as the mismatching item probe. We ensured that the different CV pairs occurred equally often inside a block as well as in each of the four serial positions. We also ensured that each memory sequence was unique over all trials.

For the mSTM tasks, four-tone sequences were presented, with pitch class distributions conforming to a familiar C major key. This was achieved by using the Krumhansl-Schmuckler key-finding algorithm (cited in [71]). Each memory sequence was correlated with the tone profiles of the 12 major and the 12 minor musical keys of the Western musical system. The highest positive correlation (also known as the maximum key correlation) provides an estimation of the key most represented in a sequence. As expected, the highest averaged correlation across all sequences within each block was with the C major tone profile (no-interference item condition: M = .69, SD = .08; no-interference order condition: M = .70, SD = .06; rhythmic interference item condition: M = .68, SD = .07; rhythmic interference order condition: M = .71, SD = .07). We ensured that all the melodic sequences used during the task were unique.

Concerning the rhythmic interference task, the sequences were constructed in the same way as described in Experiment 1.

#### Procedure

**Fig 4:**
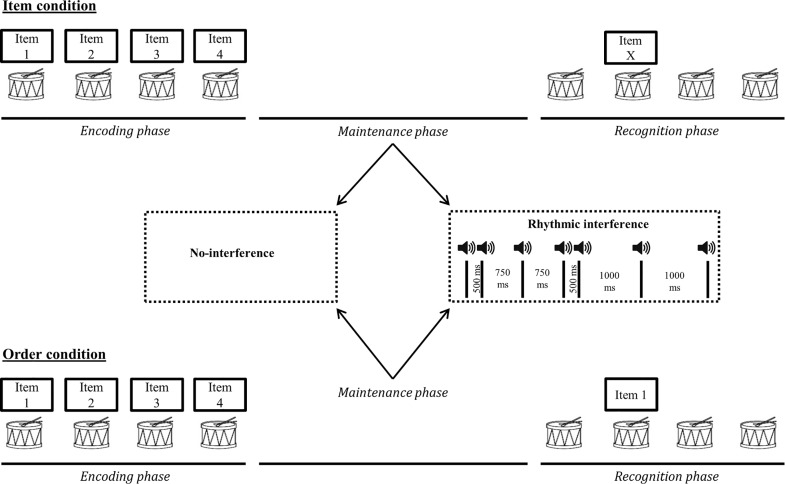
Graphical representation of task design for Experiment 2. The figures depict examples of non-matching probes for each STM task condition (i.e. item and order). Note that the same task setup characterized matching probe trials. All the task conditions (verbal *versus* musical and item *versus* order) were performed under no-interference or rhythmic tapping interference conditions.

The stimuli were presented to the participants through headphones connected to a mobile workstation at a comfortable sound level. As shown in Fig 4, the items within each sequence were presented with a 550-millisecond inter-onset-interval and were played synchronously with a regular metronome beat; note that the same beat was used for the encoding/recognition phase and the rhythmic interference, but they differed in terms of timbral information to avoid confusion. The presentation of the memory sequences was followed by a maintenance phase of five seconds. After the maintenance phase, a blue circle appeared on the center of the screen for 1100 milliseconds to announce the beginning of the recognition phase. After removal of the circle, the metronome beat was played again and participants were asked to covertly recall the sequence in time with the beat. A single item was played at a specific beat position during the recognition phase and the participants had to decide whether, the item was present or not in the target sequence (item condition), or played at the correct position or not (order condition), by pressing the corresponding response button.

Half of the trials were matching trials. For the vSTM task, a mismatching probe was a syllable not presented in the target sequence (item condition) and therefore differing from its target by both the consonant and the vowel, or a syllable presented in the target sequence but appearing in a different position than in the memory sequence (order condition); as already noted earlier, only adjacent serial position exchanges were used in this experiment. For the mSTM tasks, the mismatching probe was a tone not presented in the target sequence (item condition), or a tone presented in a different serial position than in the memory sequence (order condition). For the item condition in the mSTM tasks, the mismatching probe deviated by one tone from the target tone relative to the C major key (e.g., C could be replaced by D, or E could be replaced by either D or F) and the deviating tone was different from all other tones in the target sequence. For example, for a target sequence D-C-E-G, D was never presented as a non-matching probe for target stimuli C or E. We further ensured, for the order mSTM condition, that a negative probe could not be rejected due to the detection of a large difference in terms of pitch height between the single probe and the tones of the target sequence, by controlling the pitch changes occurring after a serial position exchange [72]. For example, for the target sequence C-G-F-A, G could be transposed in a mismatching trial to the third serial position, leading to minimal pitch changes between G and the tone initially presented at the same position (C-G-**F**-A ➔ …-…-**G**-…), but not to the first position, which would have resulted in larger pitch changes (**C**-G-F-A ➔ **G**-…-…-…).

The eight blocks of the experiment were presented in a counterbalanced order. Trials were presented randomly inside each block. In the item task conditions, participants had to decide if the probe item matched one of the items of the target sequence, independently of its serial position. For the order task conditions, participants had to judge if the probe item was presented in the same serial position as in the target sequence. In the rhythmic interference condition, the procedure was the same as in Experiment 1, except that participants were advised to stop reproducing the rhythm when they saw the blue circle appearing on the screen. Before starting each of the eight blocks, participants received specific instructions about the task requirements of the block condition.

#### Statistical Analyses

Statistical analyses followed the same procedures as in Experiment 1.

### Results

One participant showed floor-level recognition performance in the no-interference order mSTM condition (25% of correct recognitions) as well as in the interference item mSTM condition (19% of correct recognition), reflecting performance more than two standard deviations below group mean. Data of this participant were excluded from all the following analyses.

#### Recognition Performance

**Fig 5:**
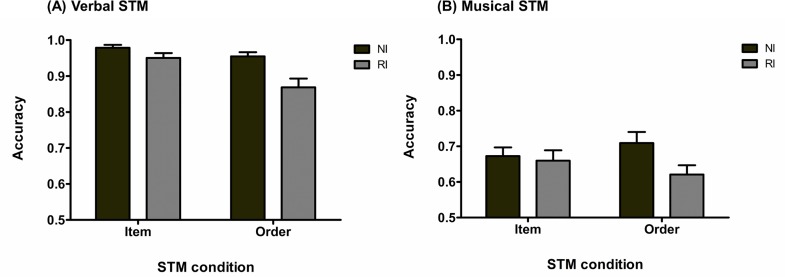
Response accuracies for Experiment 2. (A) Response accuracy (mean and standard error) for the vSTM task as a function of STM task and interference conditions (NI: no-interference; RI: rhythmic interference). (B) Response accuracy (mean and standard error) for the mSTM task as a function of STM task and interference conditions (NI: no-interference; RI: rhythmic interference).

A 2 × 2 × 2 rmANOVA on the mean proportion of correct responses showed that all main effects were significant, with a main effect of stimulus domain, *F*_(1, 28)_ = 197.58, *p* < .001, MSE = .02, η_p_^2^ = .87, of STM task condition, *F*_(1, 28)_ = 18.89, *p* < .001, MSE = .01, η_p_^2^ = .40, and of type of interference, *F*_(1, 28)_ = 4.42, *p* = .04, MSE = .01, η_p_^2^ = .14 (see Fig 5). The stimulus domain-by-STM task condition interaction, *F*_(1, 28)_ = 5.24, *p* = .03, MSE = .01, η_p_^2^ = .16, as well as the interference type-by-STM task interaction, *F*_(1, 28)_ = 13.47, *p* = .001, MSE < .01, η_p_^2^ = .32, were significant. The stimulus domain-by-type of interference interaction was not significant, *F*_(1, 28)_ = 0.08, *p* = .77, MSE = .01, η_p_^2^ < .01. Tukey’s post hoc analysis (alpha = .05) showed that rhythmic interference had a significant deleterious effect only on the order STM task condition (*p* < .001), but not on the item STM task condition (*p* = .40). Regarding the stimulus domain-by-STM task interaction, Tukey’s post hoc analysis (alpha = .05) showed no statistically significant difference between the item and order task conditions in the musical domain (*p* > .99); but performance was significantly higher for the item condition as compared to the order condition (*p* = .01) in the verbal domain. Finally, the three-way interaction was not significant, *F*_(1, 28)_ = 0.13, *p* = .72, MSE = .01, η_p_^2^ < .01.

Bayesian rmANOVA showed that, as compared to the null model, the model yielding the strongest BF was the model with the three main effects, the stimulus domain-by-STM task interaction and the interference type-by-STM task interaction (BF = 4.44E+49). The model yielding the second highest BF differed from the previous model by excluding the stimulus domain-by-STM task interaction (BF = 3.81E+49). The comparison of these two models provided only anecdotal evidence for the first model (BF = 1.16), and hence for the inclusion of the STM condition-by-type of interference interaction.

Even if the results clearly support the presence of an interaction between STM condition and interference type, we have to consider the possibility that there may have been greater sensitivity to task difficulty of memory for serial order information than of item information, this despite that within each stimulus modality performance in item and order STM conditions at baseline are fairly similar (verbal domain: no-interference item = .978, no-interference order = .955; musical domain: no-interference item: .672, no-interference order: .709).

**Fig 6:**
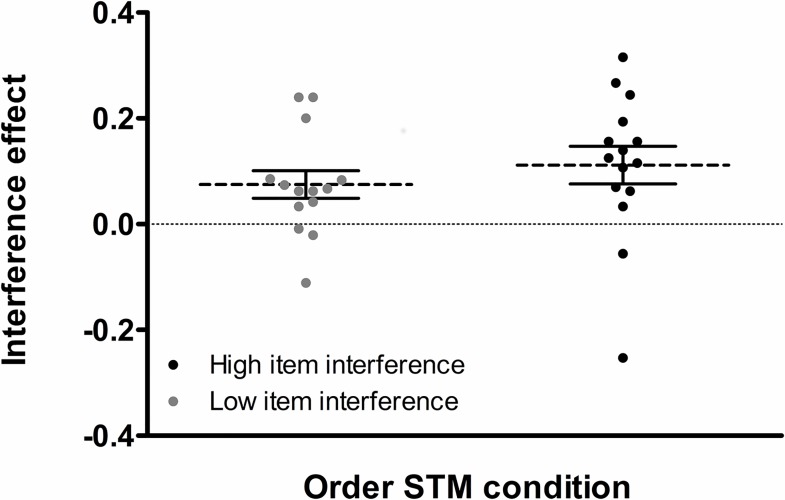
Relative effect of interference in Experiment 2. The distribution of the relative effect of interference (dashed and solid lines for means and standard errors, respectively) is shown for the order STM condition (collapsed across the two stimulus domains), as a function of item STM interference group (low item STM interference effect *versus* high item STM interference effect). Note that the two groups were determined based on a median split applied on the size of the interference effect observed in the item condition.

If the interference effects observed in the order STM condition are driven by increased sensitivity to task difficulty, then, at an interindividual level, participants being most sensitive to task difficulty in the order STM condition (i.e., those showing the largest interference effects) should also be the most sensitive to task difficulty in the item STM condition (i.e., they should also show the largest interference effects in item condition, even if these effects will be substantially smaller relative to the order STM condition), and vice-versa. There was indeed a large interindividual variability in the size of interference effects also in the item STM condition, and the range of interference effect sizes was similar in the two conditions (item interference: M = .02, SD = .11, range = -.20 to .20; order interference: M = .09, SD = .12, range = -.25 to .31; see below for details about the estimation of the size of interference effects). We checked this possibility by assessing the association of the size of interference effects between the item and order STM conditions across participants. We first determined an index of the relative amount of interference observed in each STM condition by computing the difference between recognition performance in the no-interference condition and in the rhythmic interference condition and we divided this difference by the level of recognition performance in the no-interference condition (see [36]. This measure has the advantage of being more fined grained than raw score differences by taking into account the overall level of recognition performance in the baseline condition [36]. Given the clear absence of interaction between stimulus domain and type of interference in the preceding analysis, we combined data from the two stimulus domains for each STM condition in order to optimize the reliability of the index of interference effects in the item and order STM conditions. Next, we applied a median split on the index of interference in the item condition. This allowed us to separate our sample of participants into two groups differing relative to the amount of interference caused in the item STM condition. If our results are to be explained by a greater sensitivity of the order STM condition to task difficulty more generally, then the participants showing a strong interference effect in the item STM should also show a large interference effect in the order STM condition, and thus, a median split on the item STM interference index should also separate the participants in two groups for the order STM interference index. This prediction was tested by running an independent samples *t*-test on the interference index for the order STM condition with item STM interference (high *versus* low) as group factor. As shown in Fig 6, this test showed similar levels of interference in the order STM condition for the two groups (*t*_(27)_ = 0.83, *p* = .41, Cohen’s d = 0.31); a Bayesian *t*-test provided no evidence in favor of the group effect by providing evidence for the null hypothesis but at a small level (BF = 2.21, in favor of the null model supporting an absence of effect). These results do not support an explanation of the STM condition-by-interference type interaction in terms of a higher sensitivity of the order STM condition to task difficulty since participants with the highest amount of interference in the item condition did not show larger effects of interference in the order condition.

#### Response Latencies

**Fig 7:**
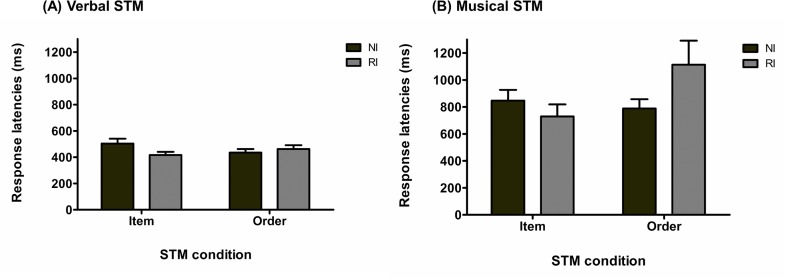
Response latencies for Experiment 2. (A) Response latencies (mean and standard error) for the vSTM task as a function of STM task and interference conditions (NI: no-interference; RI: rhythmic interference). (B) Response latencies (mean and standard error) for the mSTM task as a function of STM and interference conditions (NI: no-interference; RI: rhythmic interference).

Since two participants had RL higher than three standard deviations above the group mean in the verbal condition and higher than three standard deviations above the group mean in the musical condition, respectively, they were discarded from the subsequent analyses. As shown in Fig 7, a 2 × 2 × 3 rmANOVA performed on the mean of the median RL for correct responses across participants revealed a main effect of stimulus domain, *F*_(1, 26)_ = 25.22, *p* < .001, MSE = 4.89E+5, η_p_^2^ = .49, as well as a significant STM task condition-by-type of interference interaction, *F*_(1, 26)_ = 7.32, *p* = .01, MSE = 1.16E+5, η_p_^2^ = .22. All the remaining main effects and interaction were not significant (Type of interference: *F*_(1, 26)_ = 1.54, *p* = .22, MSE = 3.99E+5, η_p_^2^ = .06; STM task condition: *F*_(1, 26)_ = 1.57, *p* = .22, MSE = 1.29E+5, η_p_^2^ = .06; stimulus domain-by-type of interference: *F*_(1, 26)_ = 2.71, *p* = .11, MSE = 2.07E+5, η_p_^2^ = .09; stimulus domain-by-STM task condition: *F*_(1, 26)_ = 1.45, *p* = .24, MSE = 1.41E+5, η_p_^2^ = .05; three-way interaction: *F*_(1, 26)_ = 0.60, *p* = .44, MSE = 1.16E+5, η_p_^2^ = .02). Tukey’s post hoc analysis (alpha = .05) on the STM task condition-by-type of interference interaction showed that RL were significantly slowed in the order condition with rhythmic interference as compared to no-interference (*p* = .008), while no effect of interference was observed for the item condition (*p* = .99).

Complementary Bayesian rmANOVA provided a slightly different picture. The model with the highest BF was the model containing only the main effect of stimulus domain (BF = 4.19E+9), which is preferred by a factor of 2.67 over the second model with the highest BF that contains the two main effects of stimulus domain and STM task condition. These results differ from the frequentist analysis by not including the STM task condition-by-type of interaction in the model with the highest BF and by principally favoring a model with only the effect of stimulus domain. Default Bayesian analyses include interactions in a model only if the corresponding sub-effects are also included. Therefore, a model including the interaction of interest, i.e. STM task condition-by-type of interference, must include the two main effects of type of interference and of STM task condition. One way to test separately the contribution of each main effect or interaction in explaining the data is to compute the BF for each restricted model against the full model. As reported in Morey and Rouder [67], this method can be interpreted as a test for the effect or the interaction removed from the full model. As expected, the full model was preferred over the model without the main effect of stimulus domain by a factor > 100. Concerning the STM task condition-by-type of interference, a model including this interaction was favored over a model without the interaction by a factor of 4.13. Finally, the remaining restricted models were preferred by a factor of 1.57, 2.98, 2.51, 5.47, and 2.74 (full model without type of interference-by-stimulus domain interaction, without STM task condition-by-stimulus domain interaction, without main effect of interference, and without main effect of STM task condition, respectively) over the full model.

### Discussion

Experiment 2 showed an effect of rhythmic interference limited to the serial order STM tasks, with significantly lower performance and RL for order STM tasks conducted under rhythmic interference *versus* no-interference conditions and this for both verbal and musical modalities. Regarding the musical modality, the results of Experiment 2 are the first to indicate a dissociation between item STM and serial order STM processes also in the mSTM domain.

The present results also suggest that the sequentially constrained and single probe recall/recognition procedure used in Experiment 2 is better suited to study seriation processes in mSTM, as it requires musical information to be maintained and retrieved in an analytical, tone-by-tone manner, counteracting the use of global-matching processes which are likely involved when directly comparing the memory sequence to a probe sequence of equal length and structure. Furthermore, the interference effects appeared to be specific to the order STM condition and not to stem from task difficulty effects, as there was no association at the interindividual level between the size of interference effects in the item and order STM conditions, despite similar ranges of interference effect sizes in both conditions. This is also supported by a recent study showing that item and serial order STM tasks are similarly impacted by non-sequential, attention-consuming secondary tasks [73].

## General Discussion

The present study investigated the extent to which timing-based seriation mechanisms underlie the representation of serial order information in both verbal and musical domains of STM. This was done by using an interference paradigm similar to Henson et al. [31]. We hypothesized that if similar serial order processes are recruited in verbal and musical domains, then both should show selective sensitivity to interfering tasks involving a temporal processing component. Experiment 1 failed to show selective interference for serial order maintenance conditions in both verbal and musical domains, and was characterized by the use of non-sequential visuo-spatial maintenance strategies. In contrast, Experiment 2, using a sequentially constrained hybrid recall/recognition paradigm, showed a dissociation between item-based and serial order-based STM processes across both verbal and musical domains, as evidenced by a selective rhythmic interference effect on serial order but not item STM tasks.

Firstly, to the best of our knowledge, this study is the first to tackle directly the dissociation between cognitive processes involved in item *versus* serial order maintenance in mSTM. The main result of this study suggests that item and order information can be represented separately in mSTM, in line with several models of vSTM [19–23,74,75]. At the same time, the different pattern of results obtained in Experiments 1 and 2 suggests that this dissociation is task-dependent. In Experiment 1, using a standard probe sequence recognition paradigm, many participants reported using a visual strategy, favoring global matching procedures based on the comparison of contour-based relational shape patterns of the target and probe musical sequences [72] rather than storage of separated item representations and item-serial position associations. Previous experiments in musical cognition showed that the salience of a dimension such as pitch or temporal structure can prioritize the processing of one of them at the expense of the other [76–78]. Providing full melodic context at encoding and recognition, as we did in Experiment 1, could therefore have prioritized the processing of global contour shape information, at the expense of detailed, item-based pitch processing. In Experiment 2, the participants were confronted with an isolated probe presented in a sequential structure, counteracting the use of a global memory list representation [70] and constraining participants to use serial encoding and recall strategies in mSTM, inducing more serial and analytical strategies which appear to be sensitive to interference tasks requiring the processing of temporal information. Furthermore, our results show that the specific interference by temporal stimuli on order STM as opposed to item STM tasks observed by Henson et al. [31] actually depends on the specific versions of item and order STM tasks that are used. The applicability of the results reported by Henson et al. [31] to other modalities (auditory *versus* visual) and other stimulus domains (musical *versus* verbal) depends on task design and nature of stimuli. As shown in Experiment 1, the use of nonwords instead of letters or familiar syllables led to different encoding strategies as a function of item *versus* order STM requirements, making an unambiguous comparison of results for the two STM conditions difficult. Similarly, Experiment 1 shows that the use of list probes in the musical domain is likely to lead participants to rely on visuo-spatial strategies converting auditory sequences into spatial representations.

This study is the first to directly address the question of shared cognitive mechanisms underlying serial order maintenance in vSTM and mSTM. Our results are in accordance with proposals by Burgess and Hitch [20] and Hurlstone et al. [48] claiming that serial ordering processes may not be specific to the verbal domain (see also Williamson et al. [43] for a similar proposal from the musical domain). This is also in line with Patel’s expanded OPERA hypothesis [13] arguing for a close similarity between STM resources recruited to process verbal and musical information (see also [14,15]. While we think that domain-general cognitive mechanisms support coding of serial order information in STM, in line with the results of the present study, this does not mean that mSTM and vSTM are completely overlapping cognitive functions. It is likely that item-based processes (e.g., representation of the phonological characteristics of nonwords; representation of the pitch and timbre of a tone) use specialized representational systems in close interaction with sensory processing systems, in line with many recent studies showing that storage of item information strongly depends upon the accessibility of underlying representations of stimulus features [79–81]. This is partly supported by the results of Experiment 1, showing a strong and modality-specific interference of the verbal articulatory suppression task on the verbal item STM task. However, this study did not test the selective interference of musical information on musical item STM, and hence a full test of the modality specificity hypothesis for item STM still needs to be conducted.

Although the present study did not directly address the nature of the processes that interact with the coding of serial order information in both vSTM and mSTM, the interference tasks used in the present study involved temporally organized interfering material. Henson et al. [31] used this type of interfering material to investigate the hypothesis that serial order information is coded following temporal or timing signals, in line with several recent theoretical accounts of vSTM [20,23,75]. More specifically, in their computational model, Brown et al. [23] assume that serial order information is supported by temporal oscillators, analogous to the principle of a clock, while Burgess and Hitch [20] consider that serial information is encoded via a dynamically updated episodic context signal. In addition, Hartley et al. [42] recently proposed that the encoding of serial order representations could be supported by stimulus-driven mechanisms where the occurrence of the items entrains a set of multi-oscillators sensitive to local modulations of the amplitude and the phase of the speech input. The concept of oscillators mirrors recent theoretical proposals in the musical domain, Mathias et al. [45] having suggested that neuronal oscillations support contextual organization in music performance by strengthening the associations of items to specific metrical positions within a musical sequence. However, we should also note that alternative accounts of serial order coding have been proposed (e.g., [82–85], grounding serial order coding in spatial or ordinal-numerical domains, or based on sustained recurrent network representations without making an explicit distinction between item and serial order representations (see also [86]. Further work is necessary to determine the precise mechanism(s) that support serial order coding in STM. The present data, however, suggest at the least, that these mechanisms could be domain-general and are sensitive to tasks involving the processing of time-based information.

Our findings also have implications for the assessment of mSTM. In a recent review, Müllensiefen and Wiggins [87] stated that the paradigms used in mSTM studies are dominated by recognition response designs. A few authors only developed recall paradigms. One of those was Deutsch [88] who used a musical dictation method as a recall procedure of melodic sequences. This recall procedure is however only accessible to highly trained participants, i.e. musicians. A method requiring graphical reproduction of melodic contour shape is better suited for use by non-musical expert participants [89,90]. However, given that these graphical response methods facilitate global contour-based representations of target information, this paradigm is not suitable when looking at mSTM abilities in a more analytical manner. More recently, Williamson et al. [43] proposed an interesting new method requiring the participants to reproduce a musical sequence by ticking the appropriate pitch categories (i.e., “low”, “medium”, “high”) in a visual grid. However, the number of pitch categories that could be reproduced reliably by this method appeared to be limited to three for musically untrained participants. Thus this method is suboptimal for studying reproduction of musical sequences containing a higher number of non-repeating pitch categories. For this study, we developed a STM paradigm which allows for analytical mSTM representations, while using at the same time a recognition procedure and avoiding the use of global and graphical representation of the musical sequence. This method appears to have been successful for distinguishing between item-based and serial order-based processes in mSTM. Additionally, this new task has the advantage of being doable by non-musical experts. At the same time, there clearly remains an effect of expertise, performance for the vSTM tasks remaining higher than performance for the mSTM tasks in our non-musician participants. In order to equate performance levels across domains, we could have used longer list lengths for the vSTM tasks relative to the mSTM tasks, as previously done by Williamson et al. [91]. This procedure however also has an important disadvantage as it will lead to an imbalance in terms of number of serial positions to be probed and number of trials between stimulus domains. For example, in a hypothetical example where we would use 4-tone and 7-letter length sequences, we would need 16 and 28 trials for probing each serial position at least four times, for the musical and the verbal condition, respectively. In that case, it would be difficult to compare interference effects between modalities given that the amount of information that needs to be processed and maintained and on which interference will operate is not the same between modalities. At the same time, it is important to note that our main aim was to compare the sensitivity of item and order STM processes to temporal timing-signal interference *within* verbal and musical modalities, and therefore we ensured that task difficulty was equated for item and order conditions *within* each modality. Given that the analysis of recognition accuracy in Experiment 2 provided no evidence for the existence of an interaction between type of interference and stimulus-domain, our data further suggest that the order STM tasks in the two modalities were similarly sensitive to rhythmic interference.

Finally, concerning the choice of the interfering tasks, it is noteworthy to indicate that the inclusion of a pitch-related interfering condition in the context of mSTM tasks could have been theoretically interesting, as mentioned above. This would have allowed us to assess a possible double-dissociation between item and serial order information in mSTM, by showing domain-specific interference for item but not serial order STM processes. At the same time, it would have been difficult to achieve this within the task designs presented in this study, as adding a pitch-based interfering condition would have considerably increased the duration of the experiment, and increased the risk of serious cognitive fatigue effects. Note that Schendel and Palmer [92] showed that verbal and musical suppression, respectively saying “the” and singing “la”, had similar disruptive effects on recognition for auditory presented sequences of tones or digits. However, singing “la” is very close to saying “the” at the linguistic level and both may have disrupted verbal rehearsal strategies. It is also important to note that our design did not involve a control interfering task requiring no sequential or serial processes, urging us to remain cautious about the temporal nature of the interference effects that were observed. Our study aimed at determining the applicability of the experimental design proposed by Henson et al. [31] to other presentation modalities (auditory) and stimulus domains (musical). Therefore, we used an experimental paradigm remaining as close as possible to the design developed by Henson et al. [31], which also included only interfering tasks with a sequential processing component. Future studies are required to tackle more precisely the processes distinguishing retention of item and order information in vSTM and mSTM, by using, in addition to tasks involving timing-based interference, pitch-based and language-based secondary tasks that should interfere more strongly with item than order STM conditions.

## Conclusions

The present study provides evidence for a dissociation between item and serial order information in both vSTM and mSTM. This dissociation supports the hypothesis of timing-based domain-general serial ordering processes. At the same time, this dissociation appears to be task-dependent, as it is observed only for tasks imposing analytical maintenance and response processes.

## Supporting Information

S1 Data Recognition accuracy data for Experiment 1. The row in yellow corresponds to the participant with outlier recognition performance, as described in the manuscript (VNI = verbal no-interference item; VAI = verbal articulatory suppression item; VRI = verbal rhythmic interference item; VNO = verbal no-interference order; VAO = verbal articulatory suppression order; VRO = verbal rhythmic interference order; MNI = musical no-interference item; MAI = musical articulatory suppression item; MRI = musical rhythmic interference item; MNO = musical no-interference order; MAO = musical articulatory suppression order; MRO = musical rhythmic interference order).
(XLSX)

S2 Data Response latency data for Experiment 1. The rows in orange correspond to the participants with outlier RL and the row in yellow corresponds to the participant with outlier recognition accuracy, as described in the manuscript (VNI = verbal no-interference item; VAI = verbal articulatory suppression item; VRI = verbal rhythmic interference item; VNO = verbal no-interference order; VAO = verbal articulatory suppression order; VRO = verbal rhythmic interference order; MNI = musical no-interference item; MAI = musical articulatory suppression item; MRI = musical rhythmic interference item; MNO = musical no-interference order; MAO = musical articulatory suppression order; MRO = musical rhythmic interference order).
(XLSX)

S3 Data Recognition accuracy data for Experiment 2. The row in yellow corresponds to the participant with outlier recognition accuracy, as described in the manuscript (VNI = verbal no-interference item; VRI = verbal rhythmic interference item; VNO = verbal no-interference order; VRO = verbal rhythmic interference order; MNI = musical no-interference item; MRI = musical rhythmic interference item; MNO = musical no-interference order; MRO = musical rhythmic interference order).
(XLSX)

S4 Data Response latency data for Experiment 2. The rows in orange correspond to the participants with outlier RL and the row in yellow corresponds to the participant with outlier recognition performance, as described in the manuscript (VNI = verbal no-interference item; VRI = verbal rhythmic interference item; VNO = verbal no-interference order; VRO = verbal rhythmic interference order; MNI = musical no-interference item; MRI = musical rhythmic interference item; MNO = musical no-interference order; MRO = musical rhythmic interference order).
(XLSX)
